# PPARs Integrate the Mammalian Clock and Energy Metabolism

**DOI:** 10.1155/2014/653017

**Published:** 2014-02-19

**Authors:** Lihong Chen, Guangrui Yang

**Affiliations:** The Institute for Translational Medicine and Therapeutics, Translational Research Center, University of Pennsylvania, 3400 Civic Center Boulevard, Building 421, Philadelphia, PA 19104-5158, USA

## Abstract

Peroxisome proliferator-activated receptors (PPARs) are a group of nuclear receptors that function as transcription factors regulating the expression of numerous target genes. PPARs play an essential role in various physiological and pathological processes, especially in energy metabolism. It has long been known that metabolism and circadian clocks are tightly intertwined. However, the mechanism of how they influence each other is not fully understood. Recently, all three PPAR isoforms were found to be rhythmically expressed in given mouse tissues. Among them, PPAR**α** and PPAR**γ** are direct regulators of core clock components, Bmal1 and Rev-erb**α**, and, conversely, PPAR**α** is also a direct Bmal1 target gene. More importantly, recent studies using knockout mice revealed that all PPARs exert given functions in a circadian manner. These findings demonstrated a novel role of PPARs as regulators in correlating circadian rhythm and metabolism. In this review, we summarize advances in our understanding of PPARs in circadian regulation.

## 1. Introduction

### 1.1. The PPAR Family

Peroxisome proliferator-activated receptors (PPARs) are transcription factors, belonging to the nuclear receptor superfamily, a group of proteins that are usually activated by their respective ligands and function within the cell nuclei for controlling metabolism, development, and homeostasis of the organism. PPARs heterodimerize with the retinoid X receptor (RXR) and bind to PPAR responsive element (PPRE) in the regulatory region of target genes that function in diverse biological processes, such as lipid metabolism, adipogenesis, insulin sensitivity, immune response, and cell growth and differentiation [1, 2]. PPARs also participate in the pathogenesis of a cluster of human diseases, for example, metabolic syndrome that includes insulin resistance, glucose intolerance, obesity, dyslipidemia, hypertension, atherosclerosis, and microalbuminuria [3–6].

PPARs have 3 isoforms in mammals, namely, *α*, *β*/*δ*, and *γ*. Although they share structural similarity and exhibit high homology in amino acid sequence, the three isoforms are differentially expressed among tissues [7]. In general, PPAR*α* is abundant in the liver, brown adipose tissue, heart, and kidney; PPAR*γ* is mainly enriched in the adipose tissue and PPAR*β*/*δ* is ubiquitously expressed throughout the body [8]. Their differential distributions as well as different affinities to ligands attribute to their distinct roles. PPAR ligands vary from endogenous fatty acids to industrial chemicals and pharmaceutical drugs. Synthetic PPAR*α* agonists such as fenofibrate and clofibrate are clinically proven lipid-lowering drugs [9]. A class of PPAR*γ* ligands called thiazolidinediones (TZDs), such as rosiglitazone and pioglitazone, has been introduced in clinical practice for improving glycemic control via insulin sensitization in patients with type 2 diabetes [10]. Increasing evidence also points to a potential role of PPAR*β*/*δ* activators in improving insulin resistance and dyslipidemia [11]. In addition, some PPAR ligands are also potential therapeutic agents for treating hypertension, atherosclerosis, and diabetic nephropathy [1, 12, 13]. The development and use of PPAR ligands in the past decades have greatly advanced our understanding of physiological and pathological role of PPARs and the therapeutic implication of targeting them.

### 1.2. Circadian Rhythm

Circadian rhythm is any biological process displaying endogenous and entrainable oscillations of about 24 hours. In mammals, for example, sleep-awake pattern, blood pressure and heart rate, hormone secretion, body temperature, and energy metabolism exhibit circadian oscillation. These endogenous responses have significant relevance to human health and diseases [14]. Disruption of circadian rhythm has become an exacerbating factor in metabolic syndrome [15–19]. This is exceptionally important in developed countries due to frequent shift working, exposure to artificial light, travel by transmeridian air flight, and involvement in social activities. Some reports have shown that night-shift workers exhibit a higher incidence of obesity and other aspects of metabolic syndrome [15, 20, 21]. Recently, Hatori and colleagues found that temporal restriction of calorie consumption limits mouse weight gain on a high fat diet via enhancing oscillation of clock genes and their target genes [22]. Deletion of the clock gene Bmal1 (brain and muscle aryl-hydrocarbon receptor nuclear translocator-like 1) in adipose tissue causes mice to become obese via shifting their eating behavior [23]. These evidence suggested when you eat may be as important as what you eat.

**Figure 1:**
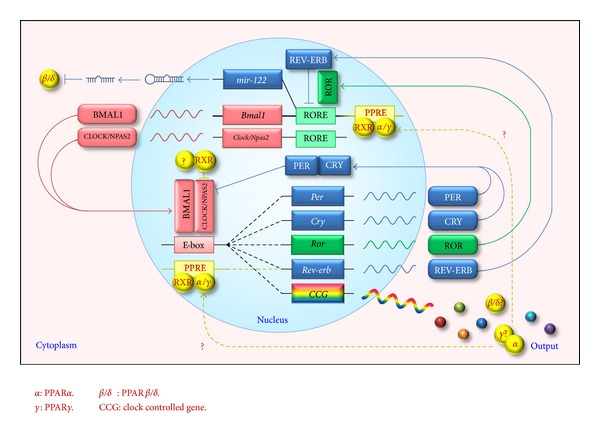
Involvement of PPARs in the transcriptional feedback loops of the mammalian circadian clock. BMAL1:CLOCK/NPAS2 heterodimer activates transcription of PER, CRY, ROR, and REV-ERB via binding to E-box in their promoters. Upon accumulation, PER and CRY dimerize and translocate into the nucleus to repress BMAL1:CLOCK/NPAS2 activity and therefore their own transcription. ROR activates and REV-ERB represses RORE-mediated transcription. These interlocking loops also control numerous output genes in a circadian manner. In addition, PPARs are integrated in this system (shown in yellow). PPAR*α* and PPAR*γ* regulate the expression of BMAL1 and REV-ERB via binding to PPRE in their promoters. PPAR*α* is also a direct target gene of BMAL1. PPAR*β*/*δ* is a target for miR-122 whose transcription is inhibited by REV-ERB. Besides, as the PPAR partner, RXR inhibits the transcriptional activity of BMAL1:CLOCK/NPAS2 complex via binding to CLOCK or NPAS2. All PPARs display circadian expression pattern in given tissues; however, it is still not known if *γ* or *β*/*δ* isoform is directly regulated by Bmal1 or if the integration of PPARs in circadian clock system forms a closed loop.

Circadian rhythm is driven by a group of genes called clock genes and has been widely observed in plants, animals and even in bacteria. In mammals, the core clock genes are rhythmically expressed in the SCN (suprachiasmatic nucleus), the master clock residing in the hypothalamus, and most peripheral tissues such as liver, fat, muscle, heart, and blood vessels [14]. These genes form a tightly regulated system with interlocking feedback and feedforward loops (Figure 1). BMAL1 and CLOCK (Circadian Locomotor Output Cycles Kaput), or its paralog NPAS2 (neuronal PAS domain protein 2), form a heterodimer. This BMAL1:CLOCK/NPAS2 complex binds to E-box elements in the promoters of Period (PER1-3) and Cryptochrome (CRY1 and CRY2) genes and activates their transcription. Upon accumulation in the cytoplasm to a critical level, the proteins of PER and CRY dimerize and translocate into the nucleus to repress the transcriptional activity of BMAL1:CLOCK/NPAS2 complex, thereby shutting down their own transcription. This core loop is interconnected with additional regulatory loops involving nuclear receptors, RAR-related orphan receptor (ROR), and REV-ERB, whose transcription is driven by BMAL1 and in turn to enhance and suppress BMAL1 transcription, respectively [24]. These feedback loops also control numerous target genes (termed clock controlled genes, CCG) in a circadian manner. Recently, all PPAR isoforms in mouse tissues were found to be rhythmically expressed [25]. Furthermore, PPAR*α* and PPAR*γ* have direct interactions with the core clock genes [26–28], suggesting that they may act as molecular links between circadian rhythm and energy metabolism.

## 2. PPAR**α** and Circadian Clock

The expression of PPAR*α* has a diurnal rhythm in mouse liver, heart, kidney, and, to a lesser extent, in the SCN [25, 29]. This circadian pattern might be partially controlled by some hormonal factors, such as glucocorticoids and insulin, whose secretions display diurnal variations [29, 30]. More recently, several reports showed a direct link between PPAR*α* and the circadian clocks. Firstly, PPAR*α* was identified as a direct target gene of BMAL1 and CLOCK via an E-box-dependent mechanism [31]. The expression level of PPAR*α* in the liver was decreased and its circadian oscillation was abolished in BMAL1 knockout mice or CLOCK-mutant mice [27, 31]. Reciprocally, PPAR*α*-null mice showed altered oscillation of BMAL1 and PER3 in the liver. *In vitro* experiments revealed that PPAR*α* directly regulates the transcription of BMAL1 and REV-ERB*α* via binding to PPRE sites in their respective promoter regions [27, 32]. Besides transcriptional regulation, PPAR*α* could modulate PER2 activity by direct physical interactions [33].

Fenofibrate, a PPAR*α* agonist and anticholesterol drug, was able to increase transcription and reset rhythmic expression of BMAL1, PER1, PER3, and REV-ERB*α* in mouse livers [27] and cultured hepatocytes [32]. Bezafibrate, another PPAR*α* agonist, was shown to phase-advance the circadian expression of BMAL1, PER2, and REV-ERB*α* in multiple mouse peripheral tissues [34, 35]. Interestingly, bezafibrate did not alter the phase of core clock genes in the SCN, although it was able to affect circadian behavior, for example, alleviating delayed sleep phase syndrome caused by clock mutation [34]. In addition, clock gene expressions were also found unaltered in the SCN of PPAR*α* knockout mice [27]. These results imply that PPAR*α* is involved in circadian regulation independently of the central clock and that PPAR*α* could be a potent target for treating sleep disorders.

More importantly, as a mediator of circadian regulation of lipid metabolism, PPAR*α* also regulates the expression of numerous genes involved in lipid and cholesterol metabolism and energy homeostasis. Many of these genes, such as sterol regulatory element binding protein (SREBP), fatty acid synthase (FAS), and HMG-CoA reductase, display daily fluctuations in mouse liver; however, their amplitudes are attenuated or abolished in PPAR*α* knockout mice [36, 37]. Kersten et al. showed that PPAR*α* mRNA is induced during fasting in wild-type mice to accommodate the increased requirement of hepatic fatty acid oxidation. PPAR*α*-null mice, however, showed a massive accumulation of lipid in their livers when fasted [38], which indicates a pivotal role of PPAR*α* in the management of energy storage during fasting. Oleoylethanolamide (OEA), an endogenous PPAR*α* agonist, is synthesized diurnally in the gut epithelium [39]. Administration of OEA produced satiety and reduced weight gain in wild-type mice, but this response was abolished in PPAR*α*-null mice [40], suggesting that PPAR*α* regulates the feeding behavior and may be a potential target for treating eating disorders.

PPAR*α* is also abundant in the heart where it serves a role in normal cardiac metabolic homeostasis via regulating enzymes involved in fatty acid beta-oxidation [41]. Its higher expression level during early dark phase is consistent with the circadian variation of heart function. Wu et al. found that both PPAR*α* and its target gene glucose transporter type 4 (Glut4) and acetyl-coA synthetase (Acs1) were significantly downregulated in the activity phase in mouse heart when cardiac tissue overexpressed PPAR*γ* coactivator 1*α* (PGC-1*α*). The disrupted circadian expression of PPAR*α* was accompanied by abolished diurnal variation of ejection fraction and shortening fraction [42], indicating that PPAR*α* plays a critical role in connecting circadian biology to heart performance.

## 3. PPAR**γ** and Circadian Clock

PPAR*γ* is a key regulator of adipogenesis and is well known for serving as a therapeutic target for treating metabolic diseases. Agonists of PPAR*γ* rosiglitazone and pioglitazone have been widely used for many years for treating type 2 diabetes, owing to their effectiveness in promoting insulin sensitivity. Recently, PPAR*γ* was shown to exhibit a remarkable circadian expression pattern in mouse liver, fat, and blood vessels [25, 28]. Global deletion of PPAR*γ* in mice abolished or dampened circadian rhythms at both behavioral and cellular levels [43].

Day-night variations in the blood pressure (BP) and heart rate (HR) are among the best known circadian rhythms of physiology. Vascular conditional deletion of PPAR*γ* in mice dampened diurnal variations of HR and BP via deregulation of BMAL1 [28]. Yang et al. found that global deletion of PPAR*γ* abolished these rhythms even without affecting locomotor activity under regular light/dark conditions [43]. In addition, pioglitazone has been shown to transform BP from a nondipper to a dipper type in type 2 diabetic patients [44]. These findings strongly support an essential role of PPAR*γ* in maintaining circadian rhythms of BP and HR, which may partially explain the beneficial side effects of PPAR*γ* agonists in cardiovascular system.

Moreover, several other key factors related to PPAR*γ* play important roles in circadian rhythm. Notably, REV-ERB*α*, a target gene of PPAR*γ* [45], is one of the core clock components, although there has not been a direct evidence showing that PPAR*γ* exerts circadian function via REV-ERB*α* yet. PPAR*γ* coactivator 1*α* (PGC-1*α*) is also identified as a circadian factor. PGC-1*α* is rhythmically expressed in mouse liver and muscle and positively regulates BMAL1, CLOCK, and REV-ERB*α* [46]. Mice lacking PGC-1*α* show abnormal locomotor activity and disrupted diurnal oscillation of body temperature and energy metabolism, which is correlated with aberrant expression pattern of metabolic genes and clock genes [46]. Nocturnin, a clock controlled gene, binds to PPAR*γ* and enhance its transcriptional activity [47]. Deletion of nocturnin abolished PPAR*γ* oscillation in the liver of mice fed on high-fat diet, accompanied by a decrease in expression of many genes related to lipid metabolism [48]. 15-Deoxy-D 12,14-prostaglandin J_2_ (15d-PGJ_2_), a natural PPAR*γ* ligand, was reported to be an entrainment factor *in vitro* [49], while its circadian function was abolished by PPAR*γ* deletion [43].

## 4. PPAR**β**/**δ** and Circadian Clock

Despite accumulating evidence supporting the role of PPAR*β*/*δ* in metabolic control and energy homeostasis [6, 50] and abundant data showing association between metabolism and circadian rhythm [51], little is known concerning the influence of PPAR*β*/*δ* in circadian rhythm unlike PPAR*α* and PPAR*γ*, even though mRNA level of PPAR*β*/*δ* is cyclic in mouse liver and brown adipose tissue [25]. REV-ERB*α* and miR-122 may serve as a possible link between PPAR*β*/*δ* and circadian clock. miR-122 is a highly abundant, liver-specific microRNA whose transcription is regulated by REV-ERB*α*. It has previously been shown to regulate lipid metabolism in mouse liver [52]. Gatfield et al. proved PPAR*β*/*δ* as a new target for miR-122, suggesting that PPAR*β*/*δ* might act as a circadian metabolic regulator in miR-122-mediated metabolic control [53]. Recently, Challet et al. observed oscillations in PPAR*β*/*δ* expression in hamster SCN. Administration of PPAR*β*/*δ* agonist L-16504 amplified the delay phase of locomotor response induced by a light pulse [54], indicating that PPAR*β*/*δ* may play a role in circadian behavior. In another recent paper, Liu et al. revealed a PPAR*β*/*δ*-dependent diurnal oscillation of *de novo* lipogenesis in mouse liver [55]. Using hepatocyte specific knockout mice, they found that PPAR*β*/*δ* controls temporal expression of hepatic lipogenic genes including acetyl-CoA carboxylase 1 (ACC1), ACC2, FAS, and stearoyl-CoA desaturase-1 (SCD1), thus affecting fatty acid metabolism in the liver.

In addition, RXR*α*, the partner of all PPARs, interacts with CLOCK protein in a ligand-dependent manner to inhibit CLOCK:BMAL1-dependent transcriptional activation of clock gene expression in vascular cells [56]. However, it is still not known whether its circadian function is dependent on PPARs. If this is the case, PPAR*β*/*δ* may be the dominant partner since *α* and *γ* isoforms were reported to be positive regulators for BMAL1 [27, 28].

## 5. Conclusions and Future Directions

Within the family of nuclear receptors, ROR and REV-ERB have been identified as core clock components. Recent findings suggest their closest phylogenetic neighbor PPARs as circadian regulators as well. PPARs have been extensively demonstrated as effective molecular targets for treating metabolic diseases. Circadian oscillations of PPARs and their target genes display a strong association with energy and metabolism homeostasis. The aberration of PPARs-circadian clock system could result in altered expression of metabolic genes, leading to disturbance in energy status. Therefore, the diseases jointly regulated by PPARs and the circadian clock have become an exploratory area. Further investigation into the regulation of PPARs in circadian rhythmic diseases could strengthen our understanding of mechanisms for disorders in energy homeostasis and metabolism and may provide novel therapeutic avenues for the treatment of metabolic ailments.

Things to keep in mind, however, most of the current studies were from mouse models. The extent to which mouse models simulate responses in human is still controversial, especially for circadian research since humans are diurnal. To date, the circadian expression of PPAR*α* in human fibroblasts [31] and PPAR*γ* in human adipose explants [57] has been identified, while how they link energy metabolism and circadian clock in humans remains to be determined.
